# Improving Recruitment and Retention of Pharmacists in a Practice-Based Research Network

**DOI:** 10.3390/pharmacy7030131

**Published:** 2019-09-04

**Authors:** Katherine Rotzenberg, Michelle A. Chui

**Affiliations:** 1 Social and Administrative Sciences Division, School of Pharmacy, University of Wisconsin-Madison, Madison, WI 53705, USA; michelle.chui@wisc.edu; 2 Sonderegger Research Center for Improved Medication Outcomes, Pharmacy Practice Enhancement and Action Research Link (PearlRx) PBRN, Madison, WI 53705, USA

**Keywords:** practice-based research network, PBRN, recruitment, retention

## Abstract

Pharmacy practice-based research networks (PBRNs) are relatively new compared to their primary care forebears, representing a unique set of research challenges. Recruitment and retention of network members are essential to maintaining the integrity of the network and achieving its research goals. Many studies have evaluated recruitment and retention of practitioners to individual studies, while far fewer have reported on recruitment and retention to the network itself. This literature review summarizes current practices for PBRN member recruitment and retention from a pharmacy perspective.

## 1. Introduction

The Agency for Healthcare Research and Quality defines practice-based research networks (PBRNs) as “groups of primary care clinicians and practices working together to answer community-based health care questions and translate research findings into practice [1]”. Although primary care PBRNs have existed in the United States since the 1970s [2], pharmacy PBRNs are relatively newer, with the majority of reports appearing in the literature over the last 20 years [3,4,5,6,7,8,9,10,11,12,13,14]. 

PBRNs provide a mechanism for practicing pharmacists to identify research questions that are relevant to their patients and translate their findings into meaningful improvement [5]. Practice-based research can establish the solid evidence base needed to support compensation for cognitive services and demonstrate the value of pharmacist interventions [4,5,13]. 

Recruitment and retention are important activities in sustaining a PBRN and helping the network achieve its goals. Recruitment is necessary throughout the lifespan of the network due to natural attrition of members through job changes [3] and to support continued growth [12]. Network growth is necessary to accommodate a larger volume of projects and to address specific topics or research questions [13]. Retention of participating and enthusiastic members contributes to the success of research projects and helps projects progress more quickly because relationships and trust have already been established.

Much of the primary care PBRN literature focuses on recruitment and retention of clinicians and practices to individual studies rather than the network itself. This literature review will summarize recruitment and retention current practices relevant to pharmacy PBRNs.

## 2. Methods

The literature search was conducted in May 2019 using the databases PubMed, SCOPUS, and Web of Science and the following search terms: “practice-based research network recruitment,” “practice-based research network retention,” “practice-based research network discontinuation,” and “practice-based research network dropout.” Articles were excluded if they only described recruitment or retention to a specific project being conducted by the practice-based research network, or if they did not describe any recruitment or retention practices. The reference lists of selected articles were reviewed for additional relevant sources.

## 3. Recruitment 

### 3.1. Characteristics of Prospective PBRN Members

PBRNs may be composed of self-selected volunteers, targeted recruits to create a representative sample, or a combination of both [15]. Recruitment of self-selected volunteers is the most common approach in both the primary care and pharmacy PBRN literature, although this does introduce a risk of bias; self-selected volunteers may provide a higher level of care or have more advanced training compared to those clinicians who choose to not participate [15]. This bias may limit the generalizability of findings to similar practice sites or clinicians [16].

Several reports from pharmacy PBRNs identify that self-selected volunteers have a strong interest in research [3,6,8,12]. Pharmacists who have previous experience with research are more likely to be interested in future research opportunities [4]. Recent pharmacy residency and clinical fellowship graduates, who have participated in research or major project management as part of their program, may be more willing to participate in future research endeavors, especially if they have completed a PGY2 residency program [11,12,17].

The conversion rate from “interested in research” to “participating in research” is low [4], so additional characteristics are useful to identify prospective PBRN members. More recent graduates (2001 and later), student preceptors, and pharmacists who are more active in making patient care recommendations are also more likely to express interest in participating in a PBRN [8]. An added benefit of recruiting student preceptors is that these pharmacists will serve as role models in research to their students, thereby encouraging a future generation of PBRN members [18]. One report describes pharmacists who work at independent community pharmacies as those who “typically want to chart their own course, look for a challenging practice opportunity, and are committed to providing quality health care in their community [6],” and thus, may be good candidates for participating in research initiatives that advance the profession and improve patient outcomes.

One successful pharmacy PBRN has reported positive relationships with pharmacies (and staff) that are “truly engaged and enthusiastic”, as well as “flexible and full of ideas [7].” In promoting PBRN membership, the PBRN should share what the network is looking for its members, as well as focus efforts on pharmacists who are most likely to participate in order to attract active, contributing members. PBRNs consisting of organization members instead of individual members may want to focus their efforts on recruiting decision-makers who can speak for the organization as a whole [7].

### 3.2. Motivators to Join a PBRN

Prospective members want to know what benefits they will receive from participation, and these should be emphasized in the recruitment process. Motivators for community pharmacists to participate in research and consider joining a PBRN are listed below:

- Opportunity to create the evidence base needed for new and sustainable pharmacy services [4,8,12].
- Personal interest in the topics being studied [4,7], and autonomy to choose participation in projects of interest [12].
- Improved professionalism and the perception of pharmacy professionalism in the community [3,4].
- Improved quality of patient care [6].
- Improved job satisfaction [3,6,12].
- Opportunity to become an APPE preceptor [6].
- Opportunity to suggest research topics [6].
- Access to clinical tools [8].
- Access to continuing education [8].

Actual benefits of PBRN participation reported by current community pharmacy PBRN members are similar to the original motivators to join the network. These include improved relationships with patients and/or healthcare providers [3,9,13], improved clinical knowledge [3], enhanced professional development [9], an enhanced relationship with the partnering school of pharmacy [9,13], improved patient care [9], improved confidence in engaging in research [9], and improved job satisfaction [13].

Compensation for network participation was cited as a positive influencer by some current pharmacy PBRN members as a gesture of goodwill; however, this approach may also create resentment among those PBRN members who are unable to receive the compensation due to company policy [3,10] and create challenges for incentivizing participation for some pharmacies [10]. A minority of independent community pharmacists (16.4%) ranked financial compensation as the most important motivator to participate in a PBRN, although the majority identified that some level of compensation was desirable [6]. Although pharmacy PBRN participation may lead to opportunities to publish and present findings [12,13], these activities are not important motivators to join a PBRN or participate in research for the majority of prospective members [4,6].

In terms of both motivators to join a PBRN and actual benefits experienced, there is a dearth of literature outside the community pharmacy setting. Two reports describe the demographics of proposed or existing pharmacy PBRNs in which the majority of members practice in acute care settings [11,14]. One report describes a PBRN of pharmacists in ambulatory primary care clinics [5]. None of these three reports describe pharmacist motivators to join the PBRN. Physicians in different subspecialties give different weights to motivators to participate in research [19]; it is possible that pharmacists in different practice settings may rank motivators differently or be interested in motivators that are not yet reported.

### 3.3. Obstacles to Joining a PBRN

Prospective PBRN members may be hesitant to join, despite the benefits, for numerous reasons. Lack of time and/or resources to participate in PBRN projects is the most commonly cited barrier across multiple reports of pharmacy PBRNs [4,6,9,12,13] and is also reported as a common barrier by other healthcare provider PBRNs [15,16]. Less frequently reported barriers include lack of experience or confidence in ability to engage in research [4,12], the belief that research activities are not compatible with the economic aspects of pharmacy [4], a perceived separation between academics and practitioners [4], a lack of perceived value of research [13], and concern for member confidentiality [10,12].

### 3.4. Strategies to Recruit PBRN Members

No recruitment strategies have been systematically studied for effectiveness within the pharmacy PBRN literature, and the list provided below identifies those strategies that have been used and reported by pharmacy PBRNs.

- Advertisement in pharmacy journal [3,12].
- Snowballing (existing members reach out to prospective members) [3,5].
- Conduct live informational outreach events located near prospective members, as well as at state professional meetings, offering continuing education if possible [7,10,12].
- Host an exhibit booth at a pharmacy conference [7].
- Contact pharmacy leaders directly [7].
- Host informational webinars, offering continuing education if possible [7,10].

Outside the pharmacy PBRN literature, successful recruitment strategies include leveraging state and national professional groups, both for their endorsement of the PBRN and access to prospective members, and obtaining testimonials from respected professional leaders [15,16,20]. Support from community advocacy and health groups can also help strengthen the appeal of the network [21]. 

Even the most robust recruitment strategy will be unsuccessful unless its messaging meaningfully connects to the prospective member. The following recruitment messaging strategies have been recommended within the pharmacy PBRN literature or by other healthcare provider PBRNs.

- Tailor the message to the prospective member by highlighting salient benefits and proactively addressing perceived barriers (as described in Section 3.2 and Section 3.3).
- Create a brand for the PBRN that clearly communicates the network’s central message [20], including its mission and vision.
- Provide examples of how research has been and can be economically beneficial to pharmacy [4], such as currently reimbursed pharmacy services that are based on previous PBRN projects.
- Describe opportunities that demonstrate member “ownership” in the PBRN and provide reassurance that the member is a valued partner [7]; this approach can help break down the perceived separation between academics and practitioners.
- Disseminate results of projects, ideally across a range of different topics, to demonstrate a history of past success and pique interest in future collaborations [13].

## 4. Retention

Although there may be excitement to participate in a PBRN initially, the busyness of everyday practice may lead to a “voltage drop” over time, and potentially, even withdrawal from the network [12,22]. In this section, strategies for maintaining engaged PBRN members over time and rationale for these strategies are discussed. Similar to PBRN recruitment strategies, no retention strategies have been systematically evaluated.

### 4.1. Communication

In the pharmacy PBRN literature, regular communication has been cited as an important component of maintaining motivation among members [3,9,13]. Effective one-way and two-way communication are vital for keeping the PBRN membership engaged, and both types of communication are regarded as essential infrastructure components for a successful network [23].

One-way communication, from the PBRN to the members, usually consists of a regular newsletter and a website in which upcoming projects are shared, results of completed projects are disseminated, and successes celebrated [3,10,12,23]. About half of pharmacy PBRN members in one study reported that a newsletter about network projects encouraged them to participate in research [3]. Patel and colleagues reported that although less than half of their pharmacy PBRN membership had visited the PBRN website and nearly three-quarters regularly read the PBRN’s newsletter, all of the PBRN members perceived communication to be adequate [9]. A newsletter can also serve as a recruitment strategy in that it can easily be distributed among prospective members to provide a snapshot of the PBRN’s recent activity. In the absence of regular communication, the network is out of sight and out of mind of those members not currently engaged in a project, even though the network may be quite active.

Two-way communication, in which members can communicate with the PBRN coordinators, and potentially other members, creates community [23] and can promote ownership of the network among the members [7]. Email listservs, message boards, blogs, conference calls or webinars, in-person meetings, and networking opportunities can all fulfill this necessary interactive component for sharing experiences and learning from others, and the exact mix of methods should be tailored to the needs of the members [4,9,12,18,23]. Among these strategies, members should be able to opt-out in order to preserve confidentiality if desired [12]. 

Advisory boards have varying functions across PBRNs, some of which can include selecting the appropriate mix of communication methods, facilitating communication, and engaging the membership [20,23].

### 4.2. Fostering Member Ownership

Members may be more willing to continue participation in the PBRN if they are more heavily invested in the network’s success. Several pharmacy and other healthcare provider PBRNs have described strategies for members to perceive more ownership of the network. Common strategies include soliciting research topics from PBRN members [4,21] and requesting feedback on the feasibility of proposed research ideas [4,10,18]. These strategies can be accomplished through a variety of two-way communication channels, as well as dedicated “project idea forms” [10]. One pharmacy PBRN engaged their members to vote on the official name of the network to promote ownership from the outset [7]. Member participation on an advisory board may be an avenue to further engage those members who are seeking leadership opportunities and further promote ownership.

### 4.3. PBRN Project Management

Member experiences with specific PBRN projects, especially the quality of two-way communication, can greatly impact whether those members consider future participation in research, separate from the PBRN’s overall communication to members [4]. Leads for specific projects within the PBRN should strive to promote member ownership in their projects by including members in the design of the project’s overall implementation from a practical standpoint [4,18,21], the design and piloting of data collection tools [3,18,21], interpreting findings [21], and supporting member presentation of findings [18,21]. It stands to reason that member engagement should lead not only to more successful project implementation, but also encourage members to participate in future projects as well.

By soliciting practical feedback from members, PBRN project leads should develop more realistic expectations of what each participating PBRN member can contribute given the constraints of their environment. PBRN members will appreciate flexibility in data collection methods to accommodate their sites, also increasing the likelihood of compliance with the project’s requirements [18]. The majority of prospective pharmacy PBRN members reported that the provision of a research assistant to help conduct the project (a recommended PBRN infrastructure element for more labor-intense projects) or a relief pharmacist, was either “important” or “very important” in their decision to participate in a PBRN [6,23]. Both of these approaches directly address the common barrier to PBRN participation of time, which monetary compensation may not adequately address. If time is available, an offer of remuneration to recognize that investment should be made, demonstrating respect for that member’s commitment even if the offer is subsequently declined [18]. In all interactions, the PBRN members should be at the center of the decision-making process [21]. Failure to recognize the burden of practice-based research upon the participating PBRN members can discourage members from participating in research [3].

## 5. Authors’ Experiences

### 5.1. Background

The Pharmacy Practice Enhancement and Action Research Link (PearlRx) is a pharmacist PBRN housed in the Sonderegger Research Center for Improved Medication Outcomes, of the University of Wisconsin-Madison School of Pharmacy, that was created in 2013. PearlRx joined forces with the Pharmacy Society of Wisconsin (the state professional society), and the other two colleges of pharmacy in the state to reinvigorate the PBRN beginning in 2018.

### 5.2. Recruitment

Between 2013–2018, recruitment efforts focused on pharmacy preceptors, specifically those who attended an annual preceptor meeting held at the school of pharmacy. Starting in 2018, recruitment efforts included the following, which substantially leveraged the strengths of our state professional society:

- Announcement of the PBRN through the state professional society’s electronic newsletter.
- Exhibits at the state professional society’s annual meeting and educational conference, with one-page handouts highlighting the benefits of membership and addressing common concerns.
- Article describing the PBRN in the state professional society’s journal.
- Continuing education presentation about PBRNs and data from a PBRN project at the state professional society’s educational conference.
- Outreach presentations to faculty and researchers at the colleges of pharmacy in the state.
- Outreach emails to pharmacy residency directors to both join the network and consider using this resource in their residency projects.
- Dissemination of membership information by the PearlRx Advisory Board to their staff.
- One-on-one discussions with pharmacy leaders and recent participants in projects.

For purposes of recruitment, a single consistent message was used for all prospective members, with the exception of pharmacy residency preceptors. For this specific audience, we created a tailored communication because this group has a set of goals that is distinctly different from other practicing pharmacists (i.e., residency accreditation standards that require resident project completion and criteria to serve as preceptors). We did not disseminate results of prior studies from the PBRN because so few had been completed at the time of the recruitment effort; as current projects are completed, their results will be added to our recruitment materials.

Our PBRN did not have the budget to support brand creation, but we were able to obtain graphic design services to create a poster, and we use consistent colors and fonts on all materials to provide a unified feel.

The result of these recruitment efforts was membership growth from 171 to 376 pharmacists, and from representation in 30 Wisconsin counties to 47 (out of 72 counties).

### 5.3. Retention 

Prior to 2018, there was no active retention strategy and almost no communication with pharmacist members. However, of the 171 members enrolled in PearlRx prior to reinvigorating the network, only eight are no longer in the network (95% retention). At least two of these are due to retirement, as the members respectfully contacted the primary author to be removed from the network. 

Starting in 2018, to strengthen a sense of community among PearlRx members and to encourage participation, a number of communication strategies were implemented, including email notifications for specific projects and a website, with a newsletter planned for the future when there are project results to disseminate. Email notifications are sent using the mail-merge feature to preserve individual confidentiality; this feature only displays the receiving member’s email address. Two-way communication includes the multiple in-person promotional events listed above, an online form for submitting project ideas for consideration, and an annual survey, all of which help foster ownership in the PBRN. In the future, we are planning to implement a member spotlight in which a PearlRx member is interviewed and featured on the website and newsletter. The use of the state professional society’s membership database as a platform for opting in and out of research network participation precludes immediate knowledge or follow-up of members who decide to leave.

## 6. Conclusions and Future Directions

Both recruitment and retention of PBRN members are essential to maintaining the integrity of the network. Recruitment strategies should include messaging—directed at the prospective member—that highlights desirable benefits and addresses perceived barriers. Retention strategies should focus on communication, fostering member ownership, and ensuring these two components continue seamlessly through individual PBRN projects. 

This literature review has uncovered several knowledge gaps which are worthy of future inquiry. Pharmacy and pharmacist PBRNs have been in a stage of infancy to adolescence and are now ready for the next phase of systematic evaluation, capitalizing on their experiences to move from primarily case report literature to more generalizable findings. Many strategies for recruitment and retention are described, and future research could help identify what strategies are most effective among pharmacists and pharmacies. Similarly, although multiple sources stress the importance of tailoring messages to specific audiences’ perceived benefits and obstacles, there are no examples in the pharmacy PBRN literature of how this is done or its degree of success among different groups. Self-determination theory, a theory of motivation which has been applied to physician PBRN members [24] and physician preceptors [25], would provide an interesting framework for classifying pharmacist motivators to join and remain in a PBRN. This approach could provide a solid theoretical underpinning for future recruitment and retention strategies.
